# Deploying triple artemisinin-based combination therapy (TACT) for malaria treatment in Africa: ethical and practical considerations

**DOI:** 10.1186/s12936-021-03649-7

**Published:** 2021-02-27

**Authors:** Paulina Tindana, Freek de Haan, Chanaki Amaratunga, Mehul Dhorda, Rob W. van der Pluijm, Arjen M. Dondorp, Phaik Yeong Cheah

**Affiliations:** 1 School of Public Health, College of Health Sciences, University of Ghana, P.O. Box LG13, Legon, Ghana; 2 Innovation Studies Group, Copernicus Institute of Sustainable Development, Utrecht University, Utrecht, The Netherlands; 3 Mahidol Oxford Tropical Medicine Research Unit, Faculty of Tropical Medicine, Mahidol University, Bangkok, Thailand; 4 Center for Tropical Medicine and Global Health, Nuffield Department of Medicine, University of Oxford, Oxford, UK

## Abstract

Malaria remains a major cause of morbidity and mortality in Africa, particularly in children under five years of age. Availability of effective anti-malarial drug treatment is a cornerstone for malaria control and eventual malaria elimination. Artemisinin-based combination therapy (ACT) is worldwide the first-line treatment for uncomplicated falciparum malaria, but the ACT drugs are starting to fail in Southeast Asia because of drug resistance. Resistance to artemisinins and their partner drugs could spread from Southeast Asia to Africa or emerge locally, jeopardizing the progress made in malaria control with the increasing deployment of ACT in Africa. The development of triple artemisinin-based combination therapy (TACT) could contribute to mitigating the risks of artemisinin and partner drug resistance on the African continent. However, there are pertinent ethical and practical issues that ought to be taken into consideration. In this paper, the most important ethical tensions, some implementation practicalities and preliminary thoughts on addressing them are discussed. The discussion draws upon data from randomized clinical studies using TACT combined with ethical principles, published literature and lessons learned from the introduction of artemisinin-based combinations in African markets.

## Background

The burden of malaria caused by *Plasmodium falciparum* has been significantly reduced worldwide since the beginning of the century but the disease still claims nearly half a million lives a year, mainly children in sub-Saharan Africa [1]. While malaria incidence and mortality has declined over the years, recent reports show that this progress has stalled. Moreover, malaria control is jeopardized, particularly in Southeast Asia, by the emergence of resistance to artemisinins and their partner drugs, resulting in decreased therapeutic efficacy of artemisinin-based combination therapy (ACT) [2, 3]. The looming threat of artemisinin and partner drug resistance spreading from Asia to Africa, or the independent emergence of resistance in Africa is particularly worrisome given that most of the malaria burden is in Africa.

Although the global pipeline for new malaria drugs in development is healthier than it has been for decades, all the most promising candidates (schizonticidals that kill the asexual blood stage of the parasite that causes the clinical manifestations of malaria) are at least five years away from being available on the market [4–6]. In the absence of new compounds, short to middle term solutions to address multidrug resistance should involve drug compounds that are currently in use. One promising direction is to add another carefully selected drug to currently deployed ACT, creating triple artemisinin-based combination therapy (TACT) to prevent the development or the spread of resistance at the population level. Since TACT is based on drugs that are currently in use they can be deployed very quickly if they are proven to be safe, well-tolerated and effective [7].

The safety and efficacy of two triple artemisinin-based combinations (dihydroartemisinin-piperaquine + mefloquine; and artemether-lumefantrine + amodiaquine) have been studied in 17 sites in Asia and 1 site in Africa [8]. The rationale for TACT is that the combination of a short-acting artemisinin with two long acting-partner drugs would make parasites less likely to encounter only one long-acting partner drug at any one time, minimizing the chance of the development of resistance. In addition, TACT is effective even in artemisinin- and multidrug-resistant infections and these triple therapies could exploit potential inverse relationships between the parasite molecular resistance mechanisms to the paired long-acting partner drugs [8, 9].

TACT might soon be one of the last remaining options using currently available drugs for effective treatment of falciparum malaria in the Greater Mekong Subregion in Asia, where treatment failure with ACT has become prominent [3, 10]. In Africa, where ACT is currently still effective, the deployment of TACT could very likely mitigate the risks of future ACT failure. However, several ethical and practical issues must be addressed prior to wide scale deployment of TACT on the African continent. Some of the most important ethical and practical considerations, and preliminary thoughts about ways these might be addressed are presented in the following sections. Data from recently completed randomized clinical studies using TACT, established ethical principles, published literature and lessons learned from the introduction of ACT in African markets are drawn upon. Regulatory and market related issues involved in a prospective transition to TACT in Africa will not be discussed in detail, as these are country and context specific.

## Ethical and practical issues of deploying TACT

The main ethical issues related to deploying TACT in Africa can be considered in terms of the risk–benefit balance, paediatric clinical ethics, public health ethics and individual autonomy. These are recurring themes in many public health initiatives. Resource allocation, sustainable use of artemisinin, affordability and market positioning of TACT in so far as it relates to ethical considerations of deploying TACT is discussed.

## *Increased risks to current patients vs benefits to future generations*

TACT is expected to be most effective at sustaining reductions in malaria-related morbidity and mortality in areas where resistance has not yet developed to any of the components, i.e. in most of sub-Saharan Africa. The long-acting partner drugs are envisioned to protect each other from the development of resistance to either of the partner drugs and protect the short acting artemisinin component [7, 8], thus reducing the chances of the emergence of multi-drug resistance and the consequent increase in illness and death. Hence the areas where they will be most effective at preventing or at least delaying the emergence of multi-drug resistance, and hence increased malaria burden, will be the ones where currently ACT remains highly effective at the individual level.

The use of TACT in Africa differs from other examples of using combination therapy in that the objective is to prevent anti-malarial drug resistance at the population level rather than at the level of the individual patient. Unlike in chronic infections, such as TB and HIV, development of de novo resistance to both components within an individual patient during treatment is rare [11]. Hence individuals could be exposed to the potential additional side effects of three rather than two drugs for little or no additional benefit to themselves. However, the two triple artemisinin-based combinations that have been tested were overall very safe and well-tolerated [8]. Incidence of vomiting during the first hour of treatment with both TACT were low, but as expected, adding mefloquine or amodiaquine to the existing artemisinin-based combinations was associated with a slight increase in the incidence of vomiting. Addition of amodiaquine slightly prolonged the QTc interval (the duration of the depolarization and subsequent repolarization of the ventricles corrected for the heart rate), but not to the extent associated with cardiac arrhythmias.

This raises the moral question of whether it is ethically justifiable for current patients in Africa to take on additional risks of experiencing additional side effects commonly experienced with anti-malarials, however minor they might be, for the sake of the public good. Avoiding drug resistance implies significant benefits both for the current population as well as for future generations: prevention of malaria related mortality and morbidity, and the related social and economic costs [12]. Many public health interventions and programmes, such as anti-malarial mass drug administrations are rooted in utilitarian ethics which focuses on maximizing benefits for the greatest number of people, in this case future populations. Therefore, asking individuals to take on additional risks for the benefit of populations and future generations is not new. Competent adults can make decisions for themselves. They can decide whether or not to take triple artemisinin-based combinations that comes with additional risks. The challenge is that many of those who will be asked to take on these additional risks are paediatric patients who cannot make decisions in their own right. Their parents or guardians must make this decision on their behalf.

## *Challenging the best interest principle in paediatric medicine*

The United Nations Conventions for the Rights of the Child emphasizes that the child’s best interest is the primary consideration in all actions concerning children [13]. Many international guidelines require that clinicians and parents adhere to the best interest principle as a guide when considering the appropriateness of the specific therapy [14]. Kopelman also suggests that this should involve “selecting the option that maximizes the person’s overall good and minimizes the person’s overall risks of harm” [15]. However, with TACT, patients are potentially exposed to the additional side effects of three rather than two anti-malarial drugs for no additional immediate benefit to themselves. Conventional wisdom would argue that this would be against the best interest of the individual child to take three rather than two drugs as this would involve taking on additional risks of experiencing side effects.

Opponents of the best interest standard argue that this standard fails to take adequately into account the interests of others and hence is inadequate for public health decision-making [16]. In the case of malaria, which is both an individual and public health issue, there is a need to balance the interest of current individuals and the interests of future patients who will contract malaria. This dilemma is not unique to TACT. Other interventions, such as mass anti-malarial drug administrations in “hotspots”, face the same challenges [17]. Other counter arguments to the best interest standard is that it is subjective, biased and that it is usually unlikely that there is only one single best option for a child [14], i.e., that there can be several reasonable options. Rhodes and Holzman suggest that as long as any chosen option does not result in significant harm to the child, it is an acceptable option, and the decision of the surrogate should be respected [14]. If TACT only poses minimal additional side effects to patients—then a clinician recommending and a parent choosing TACT over ACT—is reasonable. In the absence of resistance, choosing ACT could also be considered reasonable and if Rhodes and Holzman are followed, respecting the decision of the patient/surrogate if they chose not to take the triple artemisinin-based combination or to give it to their child. Not taking TACT can only be considered unreasonable or ‘unjust’ in the presence of resistance with unavailability of other effective treatment and assuming most people are taking the triple combinations. Another challenge is that the potential population benefits of TACT could be lost if most or all people in the community are not taking them.

## *Additional side effects of TACT*

Are additional risks of TACT minimal? For this discussion, the minimal risk standard employed in the context of research with children is borrowed [18]. To protect children in research, procedures and interventions that are not administered in the medical interests of a child must be restricted. The risk threshold for these procedures is generally measured according to the concept of minimal risk. This concept is borrowed from paediatric research ethics, for TACT used in routine clinical care, because the majority of patients who will be asked to take the triple artemisinin-based combinations in Africa will be paediatric patients.

The majority of research with children falls into two broad categories–research with the *prospect of direct benefit* to participants (e.g. access to life saving drugs) and research with *no prospect of direct benefit* to participants [18]. Since TACT does not provide any *additional* direct benefit to the individual patient over ACT (in scenarios where ACT is still effective), the latter type of research is referred to for the purpose of discussion. It is widely agreed that research with *no prospect of direct benefit* to participants are only permissible (with some exceptions) if the research poses minimal risks to participants. What is minimal risk? The minimal risk threshold is widely debated but usually taken to mean risk where “the probability and magnitude of harm or discomfort anticipated in the research are not greater in and of themselves than those ordinarily encountered in daily life or during the performance of routine physical or psychological examinations or tests” [18].

## *Autonomy and understanding*

If TACT is found to be safe, tolerable, and efficacious in clinical studies, and thereafter approved by national regulatory authorities, should patients have a choice between ACT and TACT? To respect the autonomy and freedom of choice, many would argue that patients or parents in the case of children should have a choice as both ACT and TACT are equally efficacious in settings where there is no artemisinin and ACT partner drug resistance. This decision would be made by the patient or surrogate decision-maker based on available information on the risk and benefits of ACT and TACT, their own values and life experience.

However, asking each patient to make a choice between two treatments, outside the context of a clinical study is challenging and rare in clinical practice especially in low-resource settings. Having the choice in itself could create confusion, worry and mistrust in the public health system. In addition, exercising patient autonomy to choose their preferred treatment could defeat the ongoing efforts to address the potential risks of ACT failure in Africa. The potential benefits of TACT is unlikely to be realized unless all or most patients are willing to choose TACT over ACT. One approach to addressing this dilemma might be a change in national policy to make TACT the first-line treatment or for prescribers to only recommend TACT as the first option for malaria treatment, but have ACT available for those who opt out of TACT. A more drastic approach might be to phase out ACT so that they are no longer in the market, and only have the triple artemisinin-based combinations available. Drastic measures for public health are not new; they have been taken in the interest of public health, such as quarantining patients to contain dangerous contagions such as in the COVID-19 pandemic. Does the threat of artemisinin resistance in Africa warrant these measures?

The scientific rationale for the deployment TACT in Africa could appear to be complicated for a non-scientific audience. Patients, parents and healthcare staff may not understand that TACT offers the potential to delay or prevent resistance. Rather, they may assume that TACT is more efficacious than ACT for their current malaria episode. Whether or not ACT is still available as an option, this potential misunderstanding is ethically problematic, and could lead to rumours and mistrust of the public healthcare system in the long run. Extensive and tailored community and public communication and engagement is, therefore, crucial prior and during deployment of TACT. In-depth consultations with communities, such as with community advisory boards [19], and creative strategies such as using art and theatre may be necessary to explain these difficult-to-understand concepts to healthcare staff and affected communities [20–22].

## *Resource allocation and investments*

The development and implementation of new anti-malarial therapies, such as TACT, is inherently a time and resource intensive process. To develop triple artemisinin-based combinations, there will be cost of assessing their safety and efficacy, developing appropriate formulations and obtaining regulatory approvals. Some believe that the required investment and effort should instead be put into studying existing artemisinin-based combinations, such as extending treatment regimens beyond three days, instead of developing triple combinations [23].

TACT, when available, will likely be more expensive per treatment course than conventional ACT. What should the pricing strategy of TACT be? Will patients or governments in African countries have to bear the cost, or should it be a global responsibility through mechanisms like the Global Fund? Who will manufacture co-blistered or co-formulated triple artemisinin-based combinations at scale, and where will the product development funding come from? How can a viable industry be created and how can pharmaceutical companies be engaged into developing and manufacturing triple artemisinin-based combinations? What would a transition to TACT imply in terms of intellectual property rights? These and other such questions need to be addressed collaboratively with relevant stakeholders, including pharmaceutical companies, regulatory authorities, funding agencies, health ministries, and other development partners.

Any public health intervention should be ethical, well-planned and adequately resourced. As discussed in the previous section, significant time and effort must be invested to explain to patients and all levels of healthcare staff why TACT is encouraged instead of ACT, which will add to the cost of deploying TACT.

## *Trade-offs towards sustainable use of artemisinin*

Sustainability refers to the responsible use of scarce resources to ensure that future generations can continue to benefit from them. Artemisinin is a scarce resource. There are no anti-malarial compounds presently available with comparable efficacy and losing artemisinin to resistance would imply a substantial risk to global malaria control and elimination efforts [24]. At the same time, changing first-line treatment practices is a lengthy and resource intensive process [25, 26]. In the case of a prospective transition from ACT to TACT, short-term investments at the country level are required whilst benefits are for the long-term and will transcend national borders. This means that complex trade-offs need to be made by decision-makers at both global and national levels. In addition, these trade-offs are time-dependent. The recent emergence of artemisinin resistance in Rwanda could change importantly the risk–benefit and cost analyses for the implementation of TACT, and might make their deployment more urgent [27].

In the scenario of ACT resistance spreading to or emerging locally in Africa, costs have conservatively been estimated to be over 116,000 deaths per year and the overall monetary price could add up to over USD 400 million annually [12]. Although it is hard to put a number on the exact investments that are required for changing first-line treatment to TACT in Africa, it is unlikely that such costs will come anywhere close [28]. Hence, from a health-economic perspective, preventive investments to avoid resistance appears to be good value for money, even apart from the averted increase in mortality.

Changing malaria treatment practices requires the allocation of scarce resources and strong policy coordination. Encouraging is that successes have been achieved in the past. Artemisinin has for long been available as a monotherapy in Africa [29, 30], despite significant risk of recurrent infections and the susceptibility to artemisinin resistance. After years of availability of these artemisinin monotherapies, they have now successfully been removed from the markets in most African countries. Simultaneously, availability of quality-assured ACT has significantly improved [31]. Lessons learned and best practices from previous drug transitions can be used to inform strategies to facilitate the transition to next-generation anti-malarials, such as the triple artemisinin-based combinations.

## *The market positioning of TACT*

Once TACT has been proven safe and (cost) effective, its rapid and sustainable deployment can mitigate the risk of artemisinin and partner drug resistance in Africa. To achieve this, triple artemisinin-based combinations will need to become available for affordable prices to governments and patients in endemic countries. However, the trajectory towards deployment in endemic African countries is a complex one. Previous episodes of resistance have shown that the implementation and uptake of a new generation of anti-malarial drugs can be slow and challenging [25, 32].

A multitude of actors and institutions are involved in the process of changing first-line treatment practices. At the global level, the triple artemisinin-based combinations will need to be manufactured according to standards and be subjected to review by regulatory agencies [26], funders and global technical agencies. What is encouraging is that the global health landscape has become increasingly supportive for the development and uptake of new anti-malarial therapies. Institutional arrangements, such as subsidy programmes (GFATM), regulatory frameworks (WHO prequalification) and product-development partnerships (MMV) have been established at the beginning of this millenium and now contribute to this enabling environment. At the country levels, market authorization and inclusion in national guidelines are required before TACT can be deployed on the ground. Endemic countries usually follow WHO recommendations, although delays have been reported in regulatory and implementation procedures [25, 26, 32]. This is worrisome because time can be scarce under the pressure of drug resistance. Global and national decision-makers should, therefore, anticipate pro-actively on epidemiological trends to avoid similar delays once artemisinin and partner drug combinations start to fail in Africa.

Beyond regulatory and policy procedures, TACT needs to be effectively implemented and delivered to patients in need. This again has proven to be challenging in previous anti-malarial drug transitions in Africa. Challenges have, amongst others, been associated with uncoordinated stakeholders along the value chain, misalignment with institutions, and with underperforming health systems [33–35]. As a result, availability of outdated, substandard or even counterfeit therapies persist in African countries, especially in (informal) private sector markets [29, 30, 36, 37]. Introducing new therapies under these circumstances is complex and strategies should be aligned within the broader context of improving health coverage. Encouraging are the successes that have been achieved through programmatic and regulatory initiatives in Southeast Asia [38, 39]. Similar regulatory initiatives are also proposed for enhancing treatment practices for multidrug resistant tuberculosis [35, 40]. Integrating triple artemisinin-based combinations in retail and prescription practices may require job instructions and training [28], complemented with supervision and market surveillance [41].

Addressing these multi-faceted issues is complex and requires bottom-up studies that focus on the societal embedding of TACT. Such studies are required to inform policy makers about issues in the market positioning trajectory and to develop market positioning and implementation strategies accordingly. Given the heterogeneous nature of African countries and their healthcare systems, such strategies will need to be adapted to local contexts.

## Discussion and concluding remarks

The threat of artemisinin and partner drug resistance emerging in or spreading to Africa is imminent and efforts need to be made to develop effective anti-malarial treatments. The development and deployment of TACT can be a promising strategy to delay or prevent artemisinin and partner drug resistance and also to eliminate malaria from entire circumscribed populations rapidly. A large clinical trial is underway to further characterize the safety, tolerability and efficacy of TACT over ACT in Africa and Asia (ClinicalTrials.gov Identifiers: NCT03923725 and NCT03939104). An empirical study is also underway to determine the ethical acceptability and attitudes of regulatory authorities and potential prescribers and patients, and to assess the extent to which anti-malarial drug markets in African countries are ready for a transition to TACT. Additionally, modelling studies to predict the impact of deploying TACT in different scenarios are also underway. They will model the potential of TACT to delay artemisinin resistance in Africa and also model potential economic benefits. In the present paper, pertinent ethical and practical issues regarding deploying TACT in Africa, relevant to all stakeholders involved were discussed. Considering these ethical and practical issues will be critical to reach the full potential of TACT in Africa under the threat of drug resistance.

## Data Availability

No data are associated with this article.
